# Rats, Lice, and Zinsser

**DOI:** 10.3201/eid1103.AD1103

**Published:** 2005-03

**Authors:** Gerald Weissmann

**Affiliations:** * New York University, New York, New York, USA

**Keywords:** Literature, typhus, Hans Zinsser, rheumatic fever, another dimension

Like many of my colleagues in academic medicine, I caught my first whiff of science from popular books about men and microbes. By the time we had finished high school, most of us had read and often reread Paul de Kruif's Microbe Hunters; Sinclair Lewis' Arrowsmith; and Rats, Lice and History by Hans Zinsser (Figure). It's hard nowadays to reread the work of de Kruif or Sinclair Lewis without a chuckle or two over their quaint locution, but Zinsser's *raffiné* account of lice and men remains a delight. Written in 1935 as a latter-day variation on Laurence Sterne's The Life and Opinions of Tristam Shandy, Zinsser's book gives a picaresque account of how the history of the world has been shaped by epidemics of louseborne typhus. He sounded a tocsin against microbes in the days before antibiotics, and his challenge remains meaningful today: "Infectious disease is one of the few genuine adventures left in the world. The dragons are all dead and the lance grows rusty in the chimney corner. . . . About the only sporting proposition that remains unimpaired by the relentless domestication of a once free-living human species is the war against those ferocious little fellow creatures, which lurk in dark corners and stalk us in the bodies of rats, mice and all kinds of domestic animals; which fly and crawl with the insects, and waylay us in our food and drink and even in our love" (1).

**Figure:**
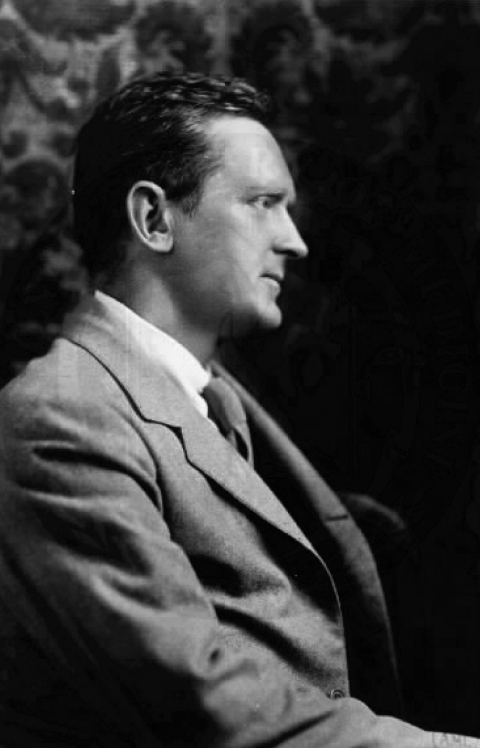
Hans Zinsser (1878–1940), author of Rats, Lice and History.

Despite the unwieldy subtitle "Being a study in biography, which, after twelve preliminary chapters indispensable for the preparation of the lay reader, deals with the life history of TYPHUS FEVER," Rats, Lice and History became an international critical and commercial success. Zinsser's romp through the ancient and modern worlds describes how epidemics devastated the Byzantines under Justinian, put Charles V atop the Holy Roman Empire, stopped the Turks at the Carpathians, and turned Napoleon's *Grand Armée* back from Moscow. He explains how the louse, the ubiquitous vector of typhus, was for most of human history an inevitable part of existence, "like baptism, or smallpox"; its habitat extended from hovel to throne. And after that Murder in the Cathedral, the vectors deserted Thomas à Becket: "The archbishop was murdered in Canterbury Cathedral on the evening of the twenty-ninth of December [1170]. The body lay in the Cathedral all night, and was prepared for burial on the following day…. He had on a large brown mantle; under it, a white surplice; below that, a lamb's-wool coat; then another woolen coat; and a third woolen coat below this; under this, there was the black, cowled robe of the Benedictine Order; under this, a shirt; and next to the body a curious hair-cloth, covered with linen. As the body grew cold, the vermin that were living in this multiple covering started to crawl out, and, as … the chronicler quoted, 'The vermin boiled over like water in a simmering cauldron, and the onlookers burst into alternate weeping and laughter …'" (1).

Zinsser's literary range and magpie intellect prompted reviewers of the day to compare him to an earlier Harvard Medical School author, Oliver Wendell Holmes. Indeed, the first few chapters of Rats, Lice and History sound a lot like Holmes' The Autocrat of the Breakfast Table. Both works offer clean drafts of political and poetic history "dipped from the running stream of consciousness"—to use Holmes' phrase, later made famous by William James. Rats, Lice and History is still in print, but it's playing to a different audience. A Yale academic worries that readers "may be puzzled by the acerbic references to the literary dandies of the interwar period" (2), while a scroll down Amazon.com yields another complaint: "Some of the writing assumes that all readers were educated under an aristocratic university system, so that there are bits thrown in Latin and Greek, not to mention French and other modern languages" (3). Be that as it may, Zinsser's assumption of an "aristocratic university system" did not prevent the book from becoming a best-seller in 1935 or from undergoing 75 subsequent printings. And as for those literary dandies: T.S. Eliot? Gertrude Stein? Lewis Mumford? Edmund Wilson?

Zinsser followed up with As I Remember Him: The Biography of R.S., a third-person autobiography that survives as a distinguished work of literature. The R.S. in the title is an abbreviation of "Romantic Self," or the last letters, inverted, of Hans Zinsser's first and last names. In it, the author spells out his warm view of medicine as a learned profession: "There is in it a balanced education of the mind and of the spirit which, in those strong enough to take it, hardens the intellect and deepens the sympathy for human suffering and misfortune" (4).

As I Remember Him was written 2 years before Zinsser's death at age 61 of lymphatic leukemia in 1940. A selection of Book of the Month Club, it reached the best-seller list as its author lay dying. News of its warm reception by book reviewers filtered into the obituaries. The volume was widely popular among doctors of my father's generation; the copy I first read sat on his office shelves, wedged between Axel Munthe's The Story of San Michele and Romain Rolland's Jean-Christophe.

As I Remember Him tells the story of Zinsser and his cohort of American physicians, who "were more fortunate than they knew, because they were about to participate in a professional evolution with few parallels" (4). Zinsser lived to see American medicine develop from a "relatively primitive dependence upon European thought to its present magnificent vigor" (4). He describes how over the years the torch of medical science passed from England to France to Germany. The flow of American medical students followed its path, which had led in central Europe to "a powerful reaction of the basic sciences upon medical training and a true spirit of research [that] pervaded medical laboratories and clinics" (4). The Golden Age of German medicine ended abruptly in 1933 when, as Zinsser lamented, "common sense became counter-revolution."

Zinsser, a New Yorker, was of German liberal stock of the 1848 generation. "The spirit of this age of my German grandfathers was one of growing philosophical materialism and the *Freie Deutsche Gemeinde* (free German communities) or 'ethical culture societies' of the sort carried on for many years by Felix Adler" (4). Zinsser's father, a free-thinking industrial chemist, raised him in a cultivated Westchester home, with trips abroad, private tutors, music, and riding lessons. He prepped for Columbia at a small private school (now the Dwight School) on 59th Street, run on ethical culture lines by Dr. Julius Sachs. At Columbia College, he fell under the spell of George Woodberry, the Mark van Doren of his day, and became enchanted by the poet and essayist who had been hailed "the American Shelley" by James Russell Lowell. Woodberry introduced Zinsser to New England enlightenment and sparked his lifelong love affair with poetry.

Zinsser's Columbia years reinforced his distrust of credulity, later strengthened by the skepticism of German enlightenment as interpreted by H.L. Mencken. Heine, the skeptic, was the Zinsser family hero, and Zinsser often repeated the poet's final words. Asked to turn to God, that He might pardon him at death, Heine had replied, "God will forgive me. It's his business." It was in this spirit that Zinsser differed from William James. "I have been utterly incapable of that 'over-belief' which William James postulates as necessary to faith. Moreover, to give religious experience—as he does—a merely pragmatic value seems both to be begging the question and to be making light of a grave problem" (4).

At Columbia, Zinsser may have fallen under the spell of Woodberry in the humanities, but in science, his mentor was Edmund B. Wilson (no relation to the writer). Zinsser vastly admired Wilson, a founder of developmental genetics in the United States. Wilson and his assistants, Zinsser knew, were the direct spiritual offspring of the Darwinian period; they had known Haeckel and Huxley. Wilson himself had described how chromosomes divide before cell cleavage and are united in pairs in the new cells. Zinsser became a convert to experimental biology after many hours in Wilson's cytology laboratories and entered Columbia's College of Physicians and Surgeons in 1903. Two of his contemporaries in the class of 1904 were his future colleagues in bacteriology, Oswald Avery, who went on to prove that bare DNA was the stuff of genes, and Joseph Thomas, father of Lewis Thomas.

Zinsser cut his teeth on research while still in medical school, earning an M.A. in embryology and studying the effects of radium on bacterial survival. (I wonder about the relationship between radium research in those early days and Zinsser's leukemia). His record earned him a prized internship at Roosevelt Hospital, then the primary teaching hospital of Columbia. Zinsser and other house officers at Roosevelt lived in a small world of their own, sharing the hazing rituals of medical training and "walking out," as dating was then called, chiefly with Roosevelt nurses, among them Grace Peck: "An interne [sic] who doesn't sooner or later fall in love with a nurse is usually a depraved fellow" (4).

The Roosevelt house officers in those days worked the wards and clinics and rode ambulances. William Carlos Williams was also an intern at Roosevelt, and his accounts of Hell's Kitchen (5) jibe with those of Zinsser. Zinsser's tale of answering a 2 a.m. call to a tenement house could be set as the beginning of a Williams poem: I found a man/Who had been shot in the chest./He was lying diagonally/across a small room/lighted by a single gas burner./He was bleeding heavily into his clothes/He also had a scalp wound/Which bled profusely./My business was to get him to the hospital . . ./I got him there alive . . . but he died (4).

Zinsser picked up at Columbia what William Carlos Williams had acquired at Penn: the spirit of pragmatic humanism that flourished in the "aristocratic" American universities of John Dewey's era. Williams' "No ideas but in things" became an anthem of the generation. Its direct predecessor was James Russell Lowell's postbellum "Commemoration Ode." Zinsser would have seen a connection between William's plea for truth in things and Lowell's ode to veritas: "No lore of Greece or Rome/No science peddling with the names of things,/Or reading stars to find inglorious fates,/Can lift our life with wings . . . /But rather far that stern device,/The sponsors chose that round thy cradle stood/In the dim, unventured wood,/The Veritas that lurks beneath/The letters unprolific sheath…." (6).

Medical schools stopped peddling with the names of things and started looking for veritas in the laboratory soon after the Flexner report of 1910. "Oh, Abraham Flexner!" Zinsser intoned, "We hail you the father—or, better the uncle—of modern medical education" (4).^1^ Flexner's exposé of the practitioner-dominated medical diploma mills that flourished at the turn of the century was the critical step in making American doctors members of a learned profession. Before then, it was all words, words, words and precious little experience; medical students sat on benches rather than working at them. After Flexner, American medical schools embarked on a century-long effort at empirical, laboratory-based medical instruction that became the envy of the world, "a phoenix rising." Zinsser would not be pleased that nowadays, as in 1933 Germany, common sense is again becoming a victim of counter-revolution. In our culture of HMOs, healthcare providers, insurance scams, and for-profit hospital chains, he'd worry that the phoenix of our youngest science might return to the "ill-smelling ashes of a big business" (4).

When their days at Roosevelt Hospital were over, William Carlos Williams became a pediatrician in Rutherford, New Jersey; Joseph Thomas entered medical practice in Flushing; and Zinsser became a microbe hunter in academia. In 1915, he accompanied the American Red Cross Sanitary Commission to investigate a devastating outbreak of typhus in Siberia. After much trial and error, he eventually succeeded in isolating the European form of the microbe that caused typhus and worked hard at developing a vaccine against it. He also moved rapidly into the new science of immunology (7).

Zinsser's scientific gifts were not limited to microbe hunting. He became a prolific medical writer and editor. His Textbook of Bacteriology went through many editions. From 1928 to 1940, he regularly published poems in the journal first edited by James Russel Lowell and named by Holmes, the Atlantic Monthly. Zinsser later explained that his cultural life bridged "the period between Emerson and Longfellow to T.S. Eliot and James Joyce" (7).

After faculty positions at Stanford and Columbia, Zinsser was appointed to the chair at Harvard in 1923. Harvard at the time was a phoenix rising and its dean, David Linn Edsall, could truly report to his trustees that "There can be little doubt that the school has acquired the standing of being the best place in the country and perhaps anywhere for advanced training in research and for advanced training of teaching and research personnel" (8).

Until the Flexnerian revolution came to Boston, indeed from the time of its founding in 1782, Harvard's Medical School had functioned chiefly as a cozy nursery for Yankee practitioners. In the days of Holmes' deanship (1847–1853), the school offered little encouragement for laboratory research to either faculty or students. In 1870, Holmes' clinical colleague James Clark White urged rigorous standards of laboratory and bedside teaching—matching those of Paris, Berlin, or Vienna—in Boston, at Harvard Medical School: "When I find the young men of Europe flocking to our shores and crowding our native students from their seats and from the bedside, when the fees of our best lecturers are mostly paid in foreign coin, and when thousands of wealthy invalids from across the sea fill the waiting-rooms of our physicians, then I will confess that I am wrong, and that of the two systems of education ours is the best. Until then I shall seek in the spirit and working of their schools the secret of their success, the cause of our failings" (9).

Its provincial air had prompted the taunt that Harvard was the best medical school in Boston. But by the 1920s, thanks to a new campus, a crop of young, full-time professors, and an ebullient Dean Edsall, innovation was in the air. Rote learning had largely yielded to learning by doing, electives had sprouted, experimental spirit had spread to the clinics, and Harvard was well on its way to becoming the best medical school in the country.

Edsall, a close friend and advisor to Flexner, assumed the deanship in 1918. He took medical education as a personal challenge and set about recasting Harvard into a world-renowned academic medical center. Frugal Edsall told of snaring Zinsser from New York, bragging that "we got this professor for $2,000 less than we could have because of [his] personal income and because he wanted to come here" (8).

The "here" to which Edsall brought Zinsser in 1926 was the neoclassic campus north of the Charles. Its open piazza was framed by buildings well-endowed with laboratory space for students and faculty. Edsall's medical acropolis attracted Zinsser, Otto Folin, Edwin Cohn, and John Edsall (the dean's son) in biochemistry; Lawrence Henderson and Walter B. Cannon in physiology; and S. Burt Wolbach and Tracy Mallory in pathology. Among the clinicians were William B. Castle, Soma Weiss, Herman Blumgart, and Maxwell Finland. Other contemporaries included future Harvard Nobelists John F. Enders, Thomas Weller, George Minot, William P. Murphy, and George Hitchings. Zinsser was in tune with Edsall's social views; despite internal opposition, the dean had appointed Alice Hamilton (1869–1970), a pioneer of industrial medicine, first woman assistant professor, not only in the medical school but in all of Harvard University (10).

Zinsser's scientific career flourished at Harvard. His work on typhus carried him to Mexico and China. In Mexico, he went after the *Rickettsia prowasekii* that caused disease, and he worked out innumerable approaches to a vaccine. He also detailed the epidemiology of a recurrent variant of typhus in European immigrants (Brill-Zinsser disease). Zinsser's work on typhus in Serbia, Mexico, and China spelled it out: lice require dirty humans, bad weather, and crowding—as in tents and barracks. That's why typhus is the stuff of war and tragedies and has, as he predicted, outlived Hans Zinsser. During World War II, typhus spread through North Africa and the Pacific Islands and devastated central Europe, where it was the second leading cause of death in German concentration camps. On or about March 31, 1945, typhus killed Anne Frank in Bergen-Belsen, only two weeks before the British Army came in to stamp out the lice (11). American troops were protected by a vaccine based on the one developed by Zinsser and Castaneda and applied on a large scale. Although epidemic typhus declined at the end of World War II with the advent of DDT (12), *R. prowasekii* is making a comeback. The largest recent outbreak since World War II was in Burundi in the mid–90s, where modern molecular techniques were used to show that a single outbreak of "jail fever" sparked an extensive epidemic of louseborne typhus in the refugee camps of Rwanda, Burundi, and Zaire—countries racked by ongoing civil war and genocide (13). There was also a brisk outbreak in Russia in 1997. In Europe in the past and Africa today, persons who "recovered" from the epidemic typhus of their youth suffer relapses of Brill-Zinsser disease and have become a reservoir of new louse-borne epidemics (12).

Work on typhus was not Zinsser's only contribution. He was also a pioneer in the study of autoimmunity, our allergy to self induced by microbes. He was drawn to its study by the leading cause of cardiac disease in the interbellum years, rheumatic fever. Zinsser had been studying allergy to the streptococcus for several years, and in 1925 published a seminal hypothesis, now accepted wisdom. Entitled "Further Studies on Bacterial Allergy: Allergic Reactions to the Hemolytic Streptococcus," it argued that, "Failure to find the organisms themselves . . . suggested either a toxic or allergic pathogenesis. Such reasoning is especially applicable to the various forms of arthritis, in which it is at least logical to think of an allergic association" (14).

We now know that "autoimmune" reactions to the microbe are responsible, but we are still in the dark as to how the disease comes about. It remained for Zinsser's student, Albert Coons to put us on the right track. Zinsser would have been pleased. In brief, Coons found that if a fluorescent molecule were chemically hooked to purified antibody, this labeled antibody could be added to samples of tissue to find the suspected antigen (15). This powerful method, immunofluorescence, was discovered by Coons and Melvin Kaplan in the 1950s to test Zinsser's suggestion that rheumatic disease is due to what might be called friendly fire: our immune defenses against the microbe are launched against our own tissues because the strep and we share look-alike components. The immune system is therefore tricked into treating the host as if it were as invader that requires disposal. Zinsser laid the groundwork for much of what we know, or think we know, of rheumatic diseases today.

Zinsser was as fine a teacher as he was a scientist. His infectious enthusiasm for pure science brought him the best and brightest students, who eventually filled chairs of bacteriology, immunology, medicine, and public health the world over and transmitted Zinsser's broad, humanistic concerns to their students. Lewis Thomas was one of those vectors, and I confess that I get twinges of what might be called a Brill-Zinsser recurrence of sentiment when I'm asked about a lifetime of teaching in medical school: "…as we grow wiser we learn that the relatively small fractions of our time which we spend with well-trained, intelligent young men are more of a privilege than an obligation. For these groups are highly selected and they force a teacher continually to renew the fundamental principles of the sciences from which his specialty takes off. So while we are, technically speaking, professors, we are actually older colleagues of our students, from whom we often learn as much as we teach them" (4).

Zinsser died of a hematologic malignancy. The last chapter of As I Remember Him forecasts in stoic detail the events of Zinsser's terminal illness. He writes of himself in the third person: "As his disease caught up with him, R.S. felt increasingly grateful for the fact that death was coming to him with due warning, and gradually. So many times in his active life he had been near sudden death by accident, violence, or acute disease…. But now he was thankful that he had time to compose his spirit, and to spend a last year in affectionate and actually merry association with those dear to him" (4).

Zinsser's legacy is indelible in the two cultures of writing and science; his well-written books remain in print, and we live with his science. He taught us how we get typhus, created a successful vaccine against it, and told us how it can recur as Brill-Zinsser disease. He was the first to reckon that rheumatic diseases result from the friendly fire of our own armaments against microbes. But, his finest contribution was to warn us, from the field, from the podium, and in his writing, that "lice, ticks, mosquitoes and bedbugs will always lurk in the shadows when neglect, poverty, famine or war lets down the defenses" (1). The shadows remain.

Dr. Weissmann is Research Professor of Medicine (Rheumatology) and Director of the Biotechnology Study Center, New York University School of Medicine. His essays and reviews have appeared in the New York Times Book Review, the London Review of Books, and the New Republic and have been collected in 7 volumes, from The Woods Hole Cantata (1985) to The Year of the Genome (2002).

## Footnotes

1 For another article on relevant topic, see the first Another Dimension published in Emerging Infectious Diseases (Myrianthopoulos NC. The philosophic origins of science and the evolution of the two cultures. Emerg Infect Dis. 2000;6:77–82. Available from http://wwwnc.cdc.gov/eid/article/6/1/00-0115_article.htm)..
